# ER-induced PERK/TFEB cascade sequentially modulates mitochondrial dynamics during cranial suture expansion

**DOI:** 10.1038/s41413-025-00427-y

**Published:** 2025-06-23

**Authors:** Jingyi Cai, Ziyang Min, Chaoyuan Li, Zhihe Zhao, Jun Liu, Dian Jing

**Affiliations:** 1 State Key Laboratory of Oral Diseases & National Center for Stomatology & National Clinical Research Center for Oral Diseases, Department of Orthodontics, West China Hospital of Stomatology, Sichuan University, Chengdu 610041, Sichuan, China, Sichuan University, Chengdu, 610041 China; 2 Department of Implantology, School and Hospital of Stomatology, Shanghai Engineering Research Center of Tooth Restoration and Regeneration, Tongji University, Shanghai, 200011 China; 3 Department of Orthodontics, Shanghai Ninth People’s Hospital, Shanghai Jiao Tong University School of Medicine, College of Stomatology, Shanghai Jiao Tong University, National Center for Stomatology, National Clinical Research Center for Oral Diseases, Shanghai Key Laboratory of Stomatology, Shanghai, 200011 China

**Keywords:** Bone quality and biomechanics, Bone

## Abstract

The effectiveness of cranial suture expansion therapy hinges on the timely and adequate regeneration of bone tissue in response to mechanical stimuli. To optimize clinical outcomes and prevent post-expansion relapse, we delved into the underlying mechanisms governing bone remodeling during the processes of suture expansion and relapse. Our findings revealed that in vitro stretching bolstered mesenchymal stem cells’ antioxidative and osteogenic capacity by orchestrating mitochondrial activities, which governed by force-induced endoplasmic reticulum (ER) stress. Nonetheless, this signal transduction occurred through the activation of protein kinase R-like ER kinase (PERK) at the ER-mitochondria interface, rather than ER-mitochondria calcium flow as previously reported. Subsequently, PERK activation triggered TFEB translocation to the nucleus, thus regulating mitochondrial dynamics transcriptionally. Assessment of the mitochondrial pool during expansion and relapse unveiled a sequential, two-phase regulation governed by the ER stress/p-PERK/TFEB signaling cascade. Initially, PERK activation facilitated TFEB nuclear localization, stimulating mitochondrial biogenesis through PGC1-α, thereby addressing energy demands during the initial phase. Subsequently, TFEB shifted focus towards ensuring adequate mitophagy for mitochondrial quality maintenance during the remodeling process. Premature withdrawal of expanding force disrupted this sequential regulation, leading to compromised mitophagy and the accumulation of dysfunctional mitochondria, culminating in suboptimal bone regeneration and relapse. Notably, pharmacological activation of mitophagy effectively mitigated relapse and attenuated bone loss, while its inhibition impeded anticipated bone growth in remodeling progress. Conclusively, we elucidated the ER stress/p-PERK/TFEB signaling orchestrated sequential mitochondria biogenesis and mitophagy under mechanical stretch, thus ensuring antioxidative capacity and osteogenic potential of cranial suture tissues.

## Introduction

Proper development of craniofacial sutures is crucial for not only the anatomical cranial and facial structures but also the proper development of hearing, vision, and neurocognitive functions.^1,2^ Orthopedic and orthodontic interventions, such as facemasks and expansion appliances, are commonly employed to address craniofacial deformities by promoting distractive osteogenesis within facial sutures.^3,4^ However, a significant proportion of patients experience substantial post-expansion relapse, primarily due to the slow and inadequate suture remodeling,^5,6^ warranting investigation into mechanisms that can enhance bone formation and remodeling during expansion therapy.

Craniofacial bones are flat bones supplemented by mesenchymal stem cells (MSCs) population within the suture during injury repair.^4^ Thus, MSCs differentiation constitutes a vital, as well as a rate-limiting step in edge bone formation upon mechanical cues.^7,8^ Given the energy-demanding nature of both cellular differentiation progress and stress response, MSC reactions to force are inherently influenced by the prevailing energy state and rely on bioenergetic metabolism to supply energy and metabolites,^9^ underscoring the importance of sustained mitochondrial activity under mechanical modulation. Noteworthy, mitochondria, as cellular powerhouses, also generate reactive oxygen species (ROS) as a byproduct of oxidative phosphorylation (OXPHOS) during ATP production.^10^ While moderate ROS levels act as “eustress” signals that initiate cell-survival mechanisms and support osteogenesis, excessive ROS production disrupts balance and leads to apoptosis.^11–13^ Consequently, the activation of antioxidant defenses, including detoxifying enzymes and mitophagy, is crucial for managing ROS and maintaining homeostasis.^14–17^ Thus, exploring how MSCs respond to the energy-demanding situation and balancing the oxidative stress in the dimension of the mitochondria may present a promising yet uncharted research avenue for revealing intricate connections between mechanical cues, bioenergetics, and bone remodeling.

One crucial structure for mitochondria to process signals is the specialized lipid raft-like domains known as mitochondria-associated membranes (MAMs) connecting the endoplasmic reticulum (ER) and mitochondria.^18^ As the site for protein synthesis and maturation, ER also serves as a key signal-transducing organelle that maintains cellular homeostasis. When exposed to cellular stress, the accumulation of unfolded protein aggregates can trigger the unfolded protein response (UPR) via transmembrane sensors on the ER. The close spatial proximity between these two organelles allows the formation of MAMs, enabling efficient signal transmission and potentially regulating mitochondrial quality.^18–20^ Mechanical forces can induce ER.^21–26^ and mitochondria alternation,^25,27–36^ with outcomes ranging from adaptive homeostasis to cellular dysfunction, depending on the type and intensity of the force. Sustained mechanical stimuli could increase markers of ER stress, triggering morphological changes such as dilatation and vesiculation, and activate the PERK-eIF2α signaling pathway.^21,23,24^ Mitochondria, in turn, undergo both morphological and quantitative changes in response to mechanical cues, including transitions between fusion and fission, as well as shifts in biogenesis and mitophagy.^25,27–36^ A recent study highlighted that ER tubules could cross over at mitochondrial to ensure the fission in responses to force.^31^ However, whether mitochondria directly perceive mechanical signals from the ER and how they respond, particularly in bone cells under stretching forces, remain largely unexplored.

In this investigation, we initially scrutinized the equilibrium between ROS and mitochondrial dynamics amidst stretching stimuli on MSCs. Subsequently, we delved into elucidating the ER-mitochondria connection, dissecting the mechanisms underlying information transmission to the mitochondria. Through in vivo examinations, we examined alterations in mitochondrial dynamics within cranial sutures throughout the expansion-relapse progression, while also elucidating the regulatory influence of ER stress and subsequent PERK/TFEB signals on these sequential changes. Finally, we interrogated the feasibility of manipulating the bone remodeling process by modulating mitochondrial pools, thereby presenting potentially efficacious solutions for clinical intervention.

## Results

### Increased mitochondrial biogenesis and mitophagy contributed to maintain the oxidation balance under stretch

**Fig. 1:**
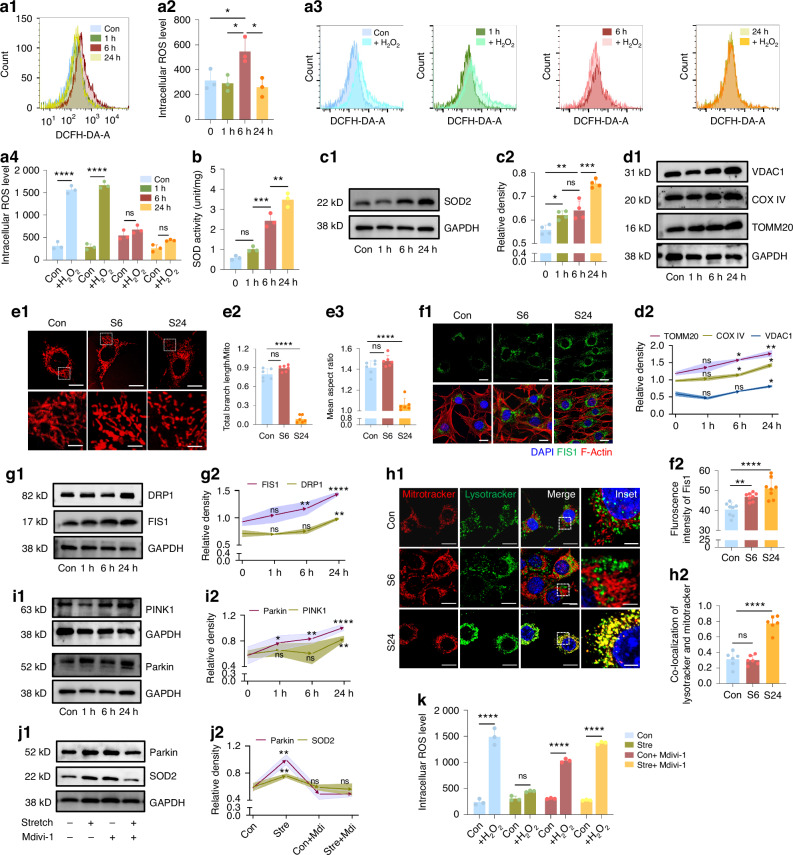
Alteration in ROS and mitochondrial dynamics in response to mechanical stretch. **a1**, **a2** Flow cytometry analysis of cellular ROS levels at different stretching points. *n* = 3/group. **a3**, **a4** ROS level changes after introducing H_2_O_2_ (final concentration 5 µmol/ml) immediately after stretch for 10 min. *n* = 3/group. **b** SOD activity changes after stretching. *n* = 3/group. **c** Western blot results of SOD2 at different stretch points. *n* = 4/group. **d** Western blot results of TOMM20, COX IV and VDAC1. Curves indicate comparisons with the control group. *n* = 3/group. **e** Live cell imaging and comparison results of Mitotracker staining. *n* = 6/group. **f** Immunofluorescence staining of FIS1. *n* = 9/group. **g** Western blot results of DRP1 and FIS1. Curves indicate comparisons with the control group. *n* = 3/group. **h** Co-localization analysis of mitochondria and lysosome (red for Mitotracker, green for Lysotracker) by live cell staining. *n* = 6/group. **i** Western blot results of PINK1 and Parkin. Curves indicate comparisons with the control group. *n* = 3/group. **j** Western blot results of Parkin and SOD2 after Mdivi application. Curves indicate comparisons with the control group. *n* = 3/group. **k** Comparison of antioxidative capacity after introducing Mdivi-1 for 24-h stretch via flow cytometry analysis of cellular ROS levels with added H_2_O_2_. *n* = 3/group. Data are presented as mean ± standard deviation (SD). ns > 0.05, *P < 0.05, **P < 0.01, ***P < 0.001, ****P < 0.000 1. Scale bar: 10 µm (low magnification), 2 µm (high magnification)

Initially, we investigated alterations of ROS in mouse C3H10T1/2 mesenchymal progenitor cells in response to mechanical stretch. Notably, ROS exhibited a biphasic pattern, with a modest peak observed after 6 h of stretch, followed by a return to baseline level after the 24-h stretch (Fig. 1a1, a2 and SI Appendix, Fig. S1a). This fluctuation prompted us to investigate whether mechanical stretching influences the cells’ ROS clearance capacity, which could account for the decline in ROS levels after 24 h. Comparative analysis revealed that cellular ROS levels remained stable despite additional H_2_O_2_ exposure in the 6-h and 24-h stretch groups, while significant increases were observed in the control and 1-h stretch groups (Fig. 1a3, a4). This finding suggests an enhanced antioxidative capacity in the 6-h and 24-h stretch groups, capable of mitigating both internally generated ROS and externally added ROS. Further examination of cellular superoxide dismutase (SOD) activity and SOD2 protein levels confirmed significant increases after 6 h of stretching, with further elevation observed at 24 h (Fig. 1b, c). Collectively, these results indicate that mechanical stimuli induce ROS production, while sustained force enhances the antioxidant response of stem cells, likely peaking at 24 h, thereby maintaining ROS homeostasis under mechanical stress.

We then scrutinized changes of nuclear factor erythroid 2-related factor 2 (NRF2), a pivotal transcription factor regulating antioxidant gene expression.^37^ Surprisingly, both the phosphorylated NRF2 (p-NRF2) ratio and its nuclear localization remained largely unchanged post-stimulation (SI Appendix, Fig. S1b, c), prompting us to explore other mechanisms involved in oxidative activity during mechanical stress. Given mitochondria’s dual roles as not only ROS producer but also contributor to antioxidative activity, we turned our attention to mitochondrial function. First, we observed an increase in mitochondrial quantity, testified by mitochondria markers as translocase of outer mitochondrial membrane 20 (TOMM20), cytochrome c oxidase IV (COX IV) and voltage-dependent anion channel 1 (VDAC1), after 6 h, with a more pronounced rise at 24 h (Fig. 1d). Second, we noted changes in mitochondrial morphology at both time points: mitochondria in the 6-h stretching group appeared slightly elongated, whereas they adopted a more rod-like shape after 24 h, suggesting a shift towards fission (Fig. 1e). This was further confirmed by elevated levels of dynamin-related protein 1 (DRP1) and fission 1 (FIS1) (Fig. 1f, g). The marked increase in mitochondrial fission likely indicated enhanced clearance, specifically through mitophagy, which was further supported by increased co-localization of mitochondria and lysosomes after 24 h of stretching (Fig. 1h) as well as the elevated markers of mitophagy, including PTEN-induced kinase 1 (PINK1) and Parkin (Fig. 1i). Inhibiting mitochondrial fission with Mdivi-1 in 24-h stretching group led to an attenuation of antioxidant capacity (Fig. 1j, k), testifying our hypothesis that mitochondria function controlled the oxidative activities and increased mitophagy contributed to the enhanced antioxidative ability following mechanical stretching.

### Enhanced ER stress as a key driver of mitophagy in response to mechanical stretch

**Fig. 2:**
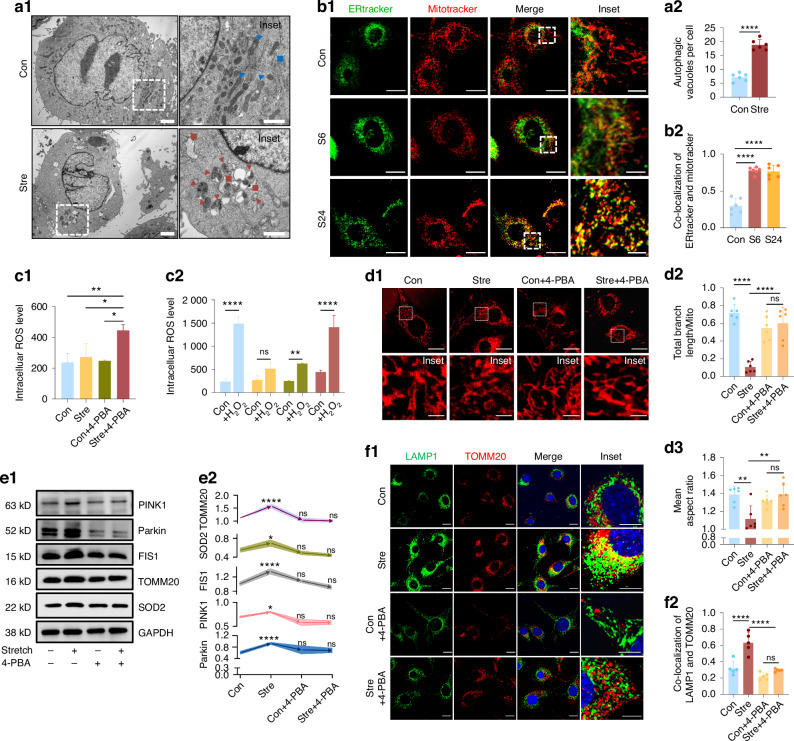
ER stress as an upstream regulator of mitophagy in response to mechanical stretch. **a** TEM images showing mitochondrial and ER changes after 24-h stretch. Blue triangles/squares: mitochondria/ER in control, red triangles/squares: mitophagy/flattened ER in stretch group. *n* = 6/group. **b** Co-localization of ERtracker (green) and Mitotracker (red). *n* = 6/group. **c** Flow cytometry analysis of ROS level after 4-PBA application. *n* = 3/group. **d** Mitochondrial morphology analysis in live cells. Scale bar: 10 µm (low magnification), 2 µm (high magnification). *n* = 6/group. **e** Western blot of SOD2, TOMM20, FIS1, Parkin, PINK1 expression after 4-PBA treatment. Curves indicate comparisons with the control group. *n* = 3/group. **f** Co-localization of LAMP1 and TOMM20 by immunofluorescence. Scale bar: 10 µm (low magnification), 2 µm (high magnification). Data presented as mean ± SD. ns > 0.05, *P < 0.05, **P < 0.01, ***P < 0.001

We started from verifying the mitochondria changes via transmission electron microscopy (TEM). Intriguingly, it appeared that after a 24-h stretch, ER became flattened and closely juxtaposed to mitochondria undergoing mitophagy (Fig. 2a), suggesting enhanced ER-mitochondria communication under mechanical stretch. Live cell imaging further confirmed increased ER-mitochondria co-localization, which became evident at 6 h and persisted up to 24 h of stretch (Fig. 2b). For changes in the ER, we observed elevated UPR, as indicated by increased phosphorylation of protein kinase R-like endoplasmic reticulum kinase (PERK) and the alpha subunit of eukaryotic translation-initiation factor 2 (eIF2α) after 24 h of stretching (SI Appendix, Fig. S2a). This was accompanied by elevated expression of transcription factor ATF4 and the spliced form of X-box-binding protein 1 (XBP1s) (SI Appendix, Fig. S2b, c). Pharmacological inhibition of ER stress using 4-phenylbutyric acid (4-PBA) significantly attenuated the stretch-induced antioxidant capacity (Fig. 2c and SI Appendix, Fig. S2d). Specifically, the 4-PBA attenuated the ROS clearance ability during stretching, causing the accumulation of ROS (Fig. 2c1, Stre vs. Stre + 4-PBA). Besides, it impaired the ability to defense external oxidative stress (Fig. 2c2, Stre + H_2_O_2_ vs. Stre + 4-PBA + H_2_O_2_). The application as well blocked mitochondria-related changes at both morphological and functional levels. Specifically, the characteristic shortening and rod-like transformation of mitochondria observed under stretch conditions were abolished (Fig. 2d). Furthermore, 4-PBA treatment prevented the elevation of mitochondrial quantity, fission, and mitophagy upon stretch loading, as evidenced by unchanged levels of TOMM20, FIS1, PINK1, and Parkin (Fig. 2e). The co-localization of lysosome/autophagosome marker lysosomal-associated membrane protein 1 (LAMP1) with mitochondrial marker TOMM20, expected to increase by mechanical stretch, was also reversed by 4-PBA treatment (Fig. 2f). These findings collectively demonstrated that elevated ER stress acted as an upstream signal driving mitochondrial change, including mitophagy, and contributed to maintaining oxidative homeostasis in response to mechanical stretching.

### P-PERK activation rather than Ca^2+^ channel activated mitophagy under stretch

**Fig. 3:**
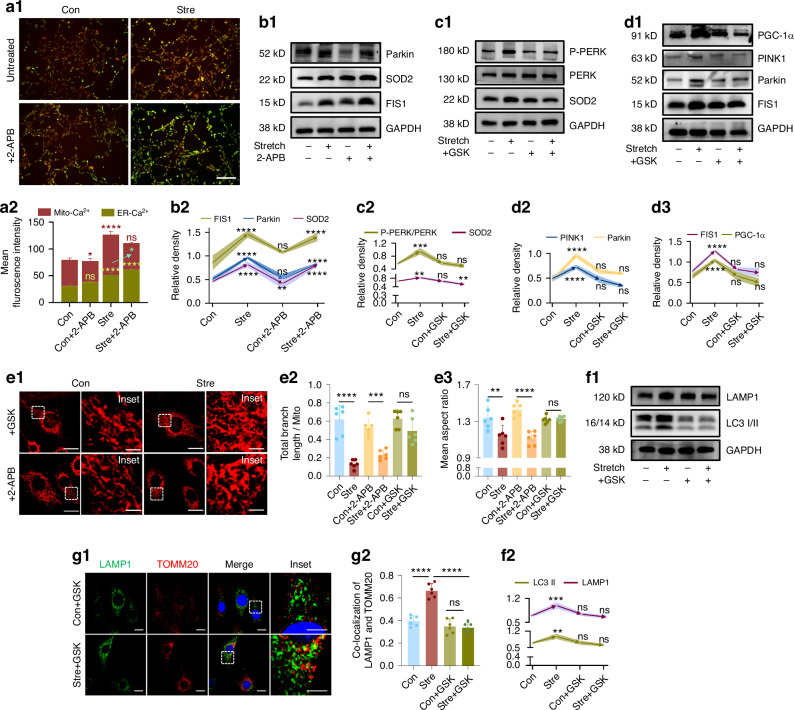
PERK phosphorylation mediated endoplasmic ER-mitochondrial communication and mitophagy in response to mechanical stretch. **a1** Live cell staining of mitochondrial calcium (red) and ER calcium (green). Scale bar: 100 µm. **a2** Comparison of fluorescence intensity of mitochondrial calcium (red) and ER calcium (green). Blue symbols indicate increased ER calcium in Stre + 2-APB vs. Stre group. *n* = 3/group. Western blot of SOD2, FIS1, and Parkin (**b**), PERK phosphorylation level and SOD2 (**c**), PINK1, Parkin, FIS1, and PGC-1α expression (**d**). Curves above indicate comparisons with the control group. *n* = 3/group. **e** Mitochondrial morphology in live cells for control and treatment groups. Scale bar: 10 µm (low magnification), 2 µm (high magnification). *n* = 6/group. **f** Western blot of LC3-II and LAMP1 expression. Curves above indicate comparisons with the control group. *n* = 3/group. **g** Co-localization of TOMM20 and LAMP1 with GSK treatment. Scale bar: 10 µm (low), 2 µm (high). *n* = 6/group. Data presented as mean ± SD. ns > 0.05, *P < 0.05, **P < 0.01, ***P < 0.001

To discern the connecting mechanism between ER and mitochondria, we first examined the calcium signal from ER to mitochondria as suggested by previous studies.^22,38,39^ Given that inositol trisphosphate receptors (IP3Rs) are the most prevalent ER Ca^2+^ channel in mitochondria–ER contact sites,^40^ we employed the IP3Rs inhibitor, 2-aminoethoxydiphenyl borate (2-APB), to assess whether impeding calcium flux would mitigate stretch-induced mitophagy. Using targeted calcium dyes specific for the ER and mitochondria, validated by co-staining with ER-Tracker and Mito-Tracker, respectively (SI Appendix, Fig. S3a, b), we observed a significant increase in mitochondrial calcium levels following mechanical stretch (Fig. 3a). This increase was effectively mitigated by the application of 2-APB. Moreover, 2-APB treatment under mechanical stretch resulted in elevated ER calcium levels, likely due to the inhibition of calcium transfer (Fig. 3a). However, 2-APB failed to inhibit the increase in SOD2 expression or the induction of mitophagy triggered by mechanical stretch (Fig. 3b). These findings suggest that although ER-mitochondria Ca^2+^ transfer occurs under mechanical stretch, it may not serve as the primary regulator of mitophagy or antioxidant capacity.

PERK is an ER stress sensor that responds to the UPR. Once activated, PERK could initiate signaling pathways to regulate ER quality control, maintain redox homeostasis, and protect mitochondrial homeostasis during stress.^41–43^ Given the specific localization of PERK on mitochondria-associated membranes (MAMs) and its well-established role in signal transduction between the ER and mitochondria,^41–44^ we conjectured whether the activation of the PERK signal under conditions of ER stress, rather than calcium flow, might serve as the upstream factor in orchestrating mitochondria changes in response to mechanical force. Using GSK2606414 (GSK), a specific PERK activation inhibitor, we found that inhibiting PERK phosphorylation reduced antioxidant capacity (Fig. 3c) and mitochondrial activity (Fig. 3d), while also preventing the expected morphological changes, such as shortening and rod-like shapes, induced by 24-h stretching (Figs. 3e and 1e1). Notably, this phenomenon was not observed with 2-APB treatment (Fig. 3b, e). This inhibition via GSK on stretch group (Stre + GSK) also abolish the expected increase of autophagic markers LAMP1 and LC3-II (Fig. 3f). It also reduced the co-localization of TOMM20 and LAMP1 comparing to Stre group (Figs. 3g and 2f1). Notably, in the absence of force, inhibiting PERK (via GSK), ER stress (via 4-PBA), or fission (via Mdivi-1) has minimal impact on cellular conditions (Figs. 3c–g, 1j, k and 2c–f). This likely reflected the naturally low levels of ER stress and mitophagy without force stimulation (Figs. 1–3). Consequently, these chemicals have little effect on antioxidative capacity under non-stimulated conditions, warranting further investigation. Collectively, these findings confirm that PERK phosphorylation, rather than Ca^2+^ transfer, mediates the signaling from the ER to mitochondria and regulates mitochondrial changes in response to mechanical stress.

### The loss of antioxidative and osteogenic ability due to relapse synchronized with the mitochondria accumulation

**Fig. 4:**
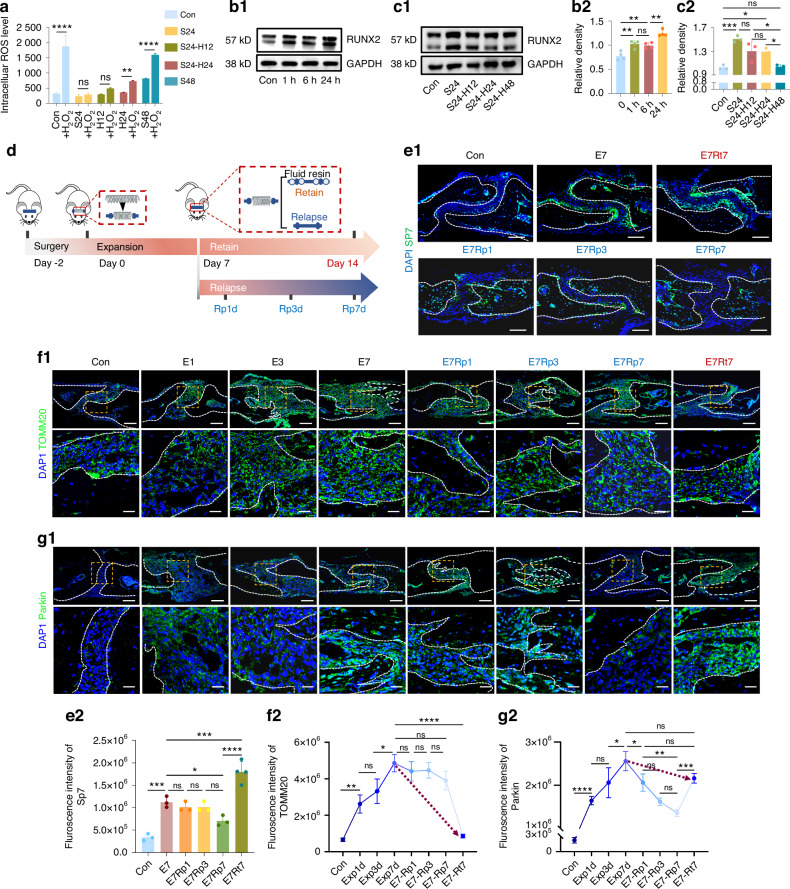
Coordinated changes in antioxidative capacity, osteogenic activity, and mitochondria during suture expansion and relapse. **a** Comparison of antioxidative capacity in in control (Con), 24-h stretch (S24), 24-h stretch with another 12-h halt (S24-H12) or 24-h halt (S24-H24), and 48-h stretch (S48) groups via flow cytometry analysis of cellular ROS levels confronting extra H_2_O_2_. *n* = 3/group. **b**, **c** RUNX2 expression analysis. *n* = 3/group. **d** Diagram of the suture expansion-relapse model. After a 7-day expansion, mice were treated with another 7-day retention (E7Rt7) or relapse for 1 (E7Rp1), 3 (E7Rp3), 7 days (E7Rp7). Immunofluorescence staining of SP7 (**e**), TOMM20 (**f**) and Parkin (**g**) in suture areas during expansion-relapse. Insets indicated by yellow squares. Red dotted lines in f2 and g2 show comparison between E7d and E7t7. Scale bar: 100 µm (low), 25 µm (high). *n* = 4–6/group. Data presented as mean ± SD. ns > 0.05, *P < 0.05, **P < 0.01, ***P < 0.001

After ensuring the anti-oxidative ability gained by stretching, we then questioned about its long-term impact, considering that mechanical force could potentially serve as ‘eustress’ to activate mitochondrial function,^45,46^ thereby boosting cellular abilities. For MSCs, this could notably refer to improved antioxidative and osteogenic capacities. Using an in vitro stretch-halt model to simulate relapse, we found that antioxidative capacity gained from 24-h mechanical stretch gradually diminished following the withdrawal of force. Specifically, this capacity was maintained for up to 12 h but significantly declined after a 24-h break, although it remained higher than in the control group (Fig. 4a) Interestingly, a 24-h stretch followed by a 24-h halt (S24-H24) showed better antioxidative ability than continuous 48-h stretching, suggesting that excessive loading may be detrimental. Regarding osteogenic ability, proper mechanical stimuli promoted osteogenesis, as indicated by increased Runt-related transcription factor 2 (RUNX2) expression after 24-h stretching (Fig. 4b). In the relapse model, RUNX2 expression mirrored the antioxidative pattern, rising during stretching and persisting for 12 to 24 h post-stretch, before returning to baseline after a 48-h break (Fig. 4c). These similar patterns indicated that while “eustress” offer benefits, these effects are limited in duration. Therefore, identifying the key transition factors responsible for the loss of these benefits may provide clinical insights.

We then proceeded with an in vivo model simulating suture expansion and relapse (Fig. 4d). After 7 days of expansion, we observed a significant increase in osteogenic potential at the suture margin, which continued to rise during the subsequent 7-day retention period. However, as relapse occurred, Sp7^+^ cells around the margin bone gradually diminished and seemed to migrate inward towards the newly formed bone. The altered localization of Sp7^+^ cells suggested that, during relapse, the osteogenic process shifts from promoting new bone formation through mesenchymal stem cell differentiation to remodeling the bone formed during the prior expansion phase (Fig. 4e). This shift in focus likely contributed to suboptimal new bone formation during relapse. The suggested benefits for mesenchymal progenitor cells gained by stretch via mitochondria prompted us to examine mitochondrial changes during this process. Specifically, mitochondrial numbers increased during expansion, peaking after 7 days, then dropped to near baseline during remodeling after retention for 7 days. In the relapse group, mitochondrial counts remained constant, potentially suggesting insufficient clearance of mitochondria (Fig. 4f). The expression of the fission marker FIS1 and the mitophagy protein Parkin as well surged in the central suture region, where predominantly housing MSCs, during the 7-day expansion, but showed contradictory patterns as to decrease during relapse (E7d to E7Rp7), while remaining high in retention groups (E7d to E7Rt7) (Fig. 4g and SI Appendix, Fig. S4a). These intriguing results suggested the concurrent loss of mitochondria clearance via mitophagy and osteogenic ability during relapse comparing to expansion. However, whether a causal relationship exists between mitochondrial imbalance and suboptimal bone generation in the cranial suture remained to be investigated.

### Temporal changes in mitochondrial biogenesis and mitophagy during cranial suture expansion and relapse

**Fig. 5:**
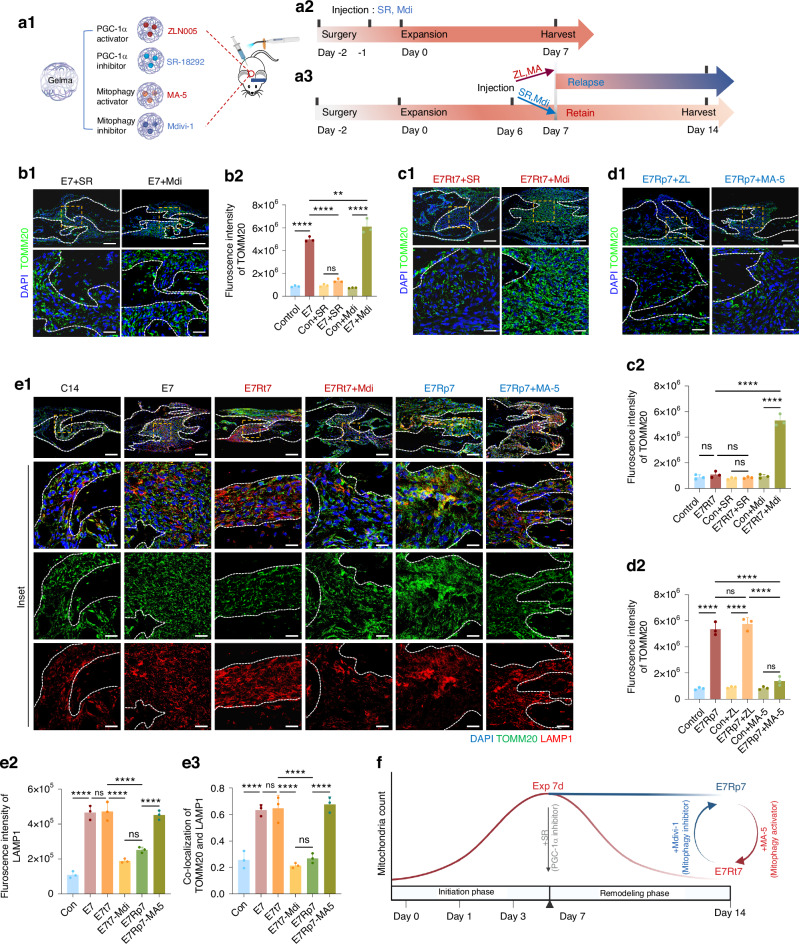
Mitochondrial dynamics during cranial suture expansion and relapse. **a** Graphical illustration of the in vivo model. **a1** Gel-MA with agents injected into the cranial suture and solidified with curing light. **a2** Diagram of pharmaceutical interventions with PGC-1α inhibitor SR-18292 (SR) or mitophagy inhibitor Mdivi-1 (Mdi) during 7-day expansion. **a3** Diagram of pharmaceutical interventions with PGC-1α activator ZLN005 (ZL) or mitophagy activator MA-5 on expansion-retention models, SR or Mdi on expansion-retention or relapse models. Immunofluorescence staining and statistical analysis of TOMM20 in the suture area after pharmaceutical interventions in 7-day expansion (**b**), 7-day expansion and 7-day retention (E7t7) (**c**), or 7-day relapse (E7Rp7) (**d**) groups. *n* = 3/group. **e1** Immunofluorescence staining of co-localization of LAMP1 and TOMM20. **e2** Statistical analysis of LAMP1 intensity. **e3** Co-localization score. *n* = 3/group. **f** Illustration of mitochondrial count changes during cranial suture expansion and relapse. SR-18292 inhibits early mitochondrial increase, Mdivi-1 prevents mitochondrial decline during retention, and MA-5 promotes mitochondrial clearance in relapse. Scale bar: 100 µm (low), 25 µm (high). Data presented as mean ± SD. ns > 0.05, *P < 0.05, **P < 0.01, ***P < 0.001

To further explore the controlling factors on mitochondria dynamics during expansion and relapse periods in vivo, we applied pharmaceutical intervention to manipulate mitophagy as well as the activity of peroxisome proliferator-activated receptor-gamma coactivator-1α (PGC-1α), the key transcription factor controlling mitochondria biogenesis.^17^ Gel-MA, a well-established hydrogel with drug-loading capabilities, was chosen to be the carrier for PGC-1α activator ZLN005 (ZL), PGC-1α inhibitor SR-18292 (SR), and mitophagy activator MA-5, as well as mitophagy inhibitor Mdivi-1, respectively (Fig. 5a1). Administering Gel-MA loaded with these agents subcutaneously into the sutural region before expansion initiation (Fig. 5a2, b) or decision-making regarding relapse or retention (Fig. 5a3, c–e) enabled us to assess the effect of mitochondrial changes.

In the initial expansion phase, SR significantly suppressed the expected increase in mitochondrial count, whereas Mdivi-1 had no effect (Figs. 5b and 4f). During the retention period, Mdivi-1 blocked the expected decline in mitochondrial count, while SR had no effect (Figs. 5c and 4f). In the relapse period, MA-5 prevented mitochondrial accumulation, rather than ZL (Fig. 5d). Furthermore, analysis of mitophagy during the retention phase confirmed the effects of MA-5 and Mdivi-1 through the mitophagy marker Parkin (SI Appendix, Fig. S5a) and the co-localization of TOMM20 and LAMP1 (Fig. 5e).

Conclusively, the results suggested that during the initial expansion phase, PGC-1α activated mitochondrial biogenesis to counteract mechanical stress. During the retention period, mitophagy assumed control to clear excessive mitochondria, resulting in the gradual reduction of total mitochondrial count. Conversely, upon relapse occurrence, the anticipated mitophagy during the remodeling process failed to activate, leading to mitochondrial accumulation (Fig. 5f).

### PERK phosphorylation orchestrated biphasic mitochondria changes during expansion

**Fig. 6:**
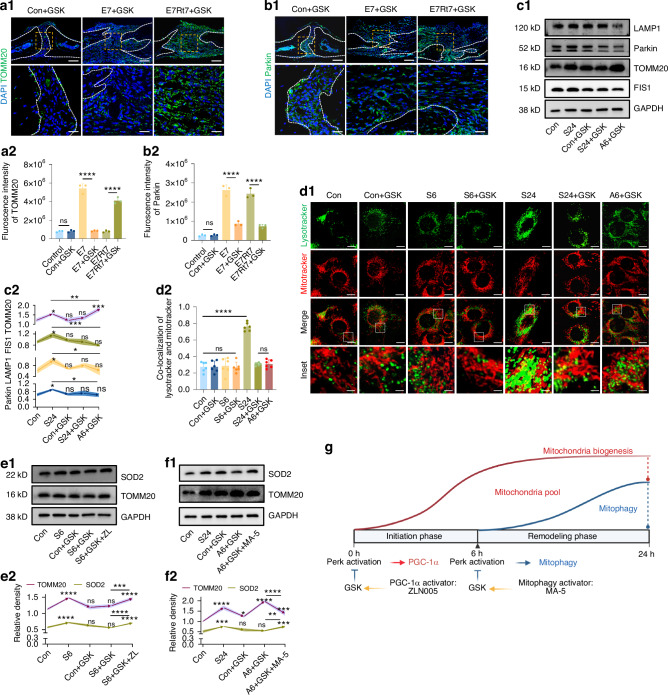
Phosphorylation of PERK directs mitochondrial changes during expansion and relapse. Immunofluorescence staining and statistical analysis of TOMM20 (**a**) and Parkin (**b**) in the suture area after GSK2606414 (GSK) treatment. Scale bar: 100 µm (low), 25 µm (high). *n* = 3/group. **c** Western blot showing TOMM20, FIS1, LAMP1, and Parkin expression in control (Con), 24-h stretch (S24) groups, as well as groups treated with GSK in control (Con + GSK) and S24 (S24 + GSK), and groups stretched for 24 h with GSK added halfway at 6-h point (A6-GSK). Curves show comparison with control group. *n* = 3/group. **d** Mitotracker and Lysotracker live cell staining (**d1**) and statistical analysis (**d2**) of mitochondria-lysosome co-localization. Scale bar: 10 µm (low), 2 µm (high). *n* = 6/group. **e** Western blot of TOMM20 and SOD2 expression in control, S6, Con + GSK, S6 + GSK, and S6 + GSK rescued with ZLN005 (S6 + GSK + ZL). *n* = 3/group. **f** Western blot of TOMM20 and SOD2 expression in control, S24, Con + GSK, A6 + GSK, and A6 + GSK rescued with MA5 (A6 + GSK + MA5). *n* = 3/group. **g** Illustration showing mitochondrial pool changes during in vitro stretching. Initial PERK phosphorylation activates PGC-1α for mitochondrial biogenesis (red line). After 6 h, PERK activates mitophagy, resulting in mitochondrial clearance (blue line). GSK treatment can be rescued by ZLN005 (initiation phase) or MA-5 (later stage). Data presented as mean ± SD. ns > 0.05, *P < 0.05, **P < 0.01, ***P < 0.001

After confirming that the mitochondrial pool underwent a rise-and-fall cycle during the expansion and retention periods, with relapse leading to mitochondrial accumulation due to impaired mitophagy, we further investigated the mechanisms by which mechanical signals were transmitted. Based on our in vitro findings, we first testified the role of ER stress signaling and PERK phosphorylation in regulating mitochondrial dynamics using Gel-MA-carried GSK injections. GSK application at the early stage hindered mitochondrial biogenesis, evidenced by the blocked rise in TOMM20 in the E7 group (Figs. 6a and 4f; E7 vs. E7 + GSK). Conversely, GSK impeded the decline in mitochondrial count during retention (Figs. 6a and 4f, E7t7 vs. E7t7 + GSK), by suppressing mitophagy, as indicated by reduced Parkin (Figs. 6b and 4g) and FIS1 (SI Appendix, Figs. S4a and S6a) levels in the E7Rt7-GSK group. The contrasting effects of GSK at different time points were further validated in vitro. In the cellular stretch model, GSK applied within the first 6 h (S6 + GSK) repressed TOMM20 protein levels (Fig. 6c). In contrast, later-stage GSK application (applied after 6-h stretch, A6 + GSK) significantly inhibited mitophagy, leading to mitochondrial accumulation during the subsequent 18 h (Fig. 6c). Live cell imaging visualized these differential effects on mitochondrial counts: continuous GSK application blocked all stretch-induced changes (S24 + GSK), whereas early application impacted mitochondrial count growth (S6 + GSK), and later application specifically blocked Lysotracker-Mitotracker co-localization which resulting accumulation of mitochondria (A6 + GSK) (Fig. 6d).

To further validate the PERK’s biphasic effect in mitochondria growth and clearance, we examine whether PGC-1α activator ZL and mitophagy activator MA-5 could counteract PERK dephosphorylation’ effect, thereby rescuing the effect of GSK application. Specifically, ZL restored mitochondrial count within the first 6 h (Fig. 6e), while MA-5 suppressed mitochondrial accumulation in the latter stage (Fig. 6f). These findings demonstrated that PERK phosphorylation orchestrated mitochondrial dynamics in two stages: initially enhancing biogenesis to meet energy demands, and subsequently activating mitophagy to maintain balance by eliminating excess mitochondria. Thus, ZL alleviated the suppressive impact of GSK in the initial 6 h, while MA-5 was effective during the following 18 h (Fig. 6g).

### PERK phosphorylation controlled TFEB nuclear translocation to direct mitochondria dynamics

**Fig. 7:**
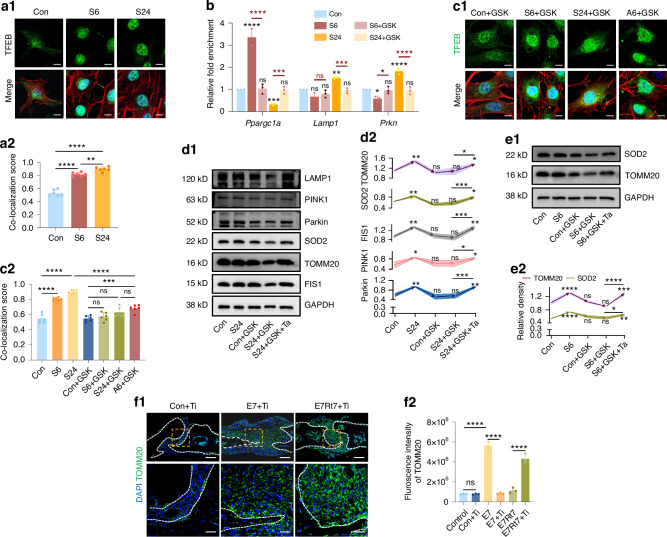
Phosphorylation of PERK promoted TFEB nuclear translocation to influence mitochondrial dynamics. **a** Cellular immunofluorescence analysis of TFEB co-localization with DAPI in control (Con), 6-h stretch (S6), and 24-h stretch (S24) groups. Scale bar: 10 µm. *n* = 6/group. **b** The relative fold enrichment of *Ppargc1α* (coding PGC-1α), *Lamp1* (coding LAMP1), and *Prkn* (coding Parkin) to Spike in DNA in CUT&RUN assay with TFEB antibody. *n* = 3/group. The black symbol on the top of column represents its comparison to control group. The red symbol represents comparison between groups. **c1** Cellular immunofluorescence images of TFEB in groups treated with GSK2606414 (GSK) in control (Con + GSK), 6-h stretch (S6 + GSK), 24-h stretch (S24 + GSK) groups, and groups stretched for 24 h with GSK added halfway after 6-h stretch (A6 + GSK). Scale bar: 10 µm. *n* = 6/group. **c2** Statistical analysis of the co-localization score of TFEB with DAPI. **d** Western-bolt analysis of Parkin, PINK1, LAMP1, FIS1, SOD2 and TOMM20 in Con, S24, Con-GSK, S24-GSK, and S24-GSK rescued with TFEB activator (S24 + GSK + Ta). Comparative labels on the curves indicate comparison with the control group. *n* = 3/group. **e** Western-bolt analysis of TOMM20 and SOD2 in Con, S6, Con + GSK, S6 + GSK, and S6 + GSK rescued with Ta (S6 + GSK + Ta) groups. Comparative labels on the curves indicate comparison with the control group. *n* = 3/group. **f** Immunofluorescence staining of TOMM20 in the suture area following GSK intervention. Scale bar: 100 µm for low magnification and 25 µm for high magnification. *n* = 3/group. Data are presented as mean ± standard deviation (SD). ns > 0.05, *P < 0.05, **P < 0.01, ***P < 0.001, ****P < 0.000 1

To understand how PERK activation exerts distinct functions in two stages, we investigated the role of the transcription factor TFEB, a downstream target of PERK,^44^ which has recently been identified as a key regulator of mitochondrial dynamics through its effects on both PGC-1α and mitophagy-related genes.^44,47,48^ To investigate this hypothesis, we first validated the increased nuclear translocalization of TFEB after 6- and 24-h stretch (Fig. 7a). Subsequently, CUT&RUN-qPCR was applied to validate the sequential transcriptional regulation role of TFEB. We observed a significant augmentation in TFEB’s binding to the *Ppargc1α* sequence after 6 h of mechanical strain, albeit diminishing below baseline levels by the 24-h mark. Conversely, TFEB’s association with *Lamp1* and *Prkn* sequence exhibited a slight decrease at 6 h, but significantly increased by 24 h. These binding changes were all blocked by GSK application (Fig. 7b). Fluorescence results indicated that PERK phosphorylation controlled the nuclear import of TFEB under stretch, as GSK blocked the expected transfer induced by stretching (Fig. 7a, c). Besides, TFEB activation (Ta) reversed the outcomes of GSK-induced PERK deactivation at both 6-h and 24-h time points (Fig. 7d, e). Furthermore, injection of TFEB inhibitor (Ti) mimicked GSK’s sequential inhibitory effects, impacting mitochondrial biogenesis in the initial phase and mitophagy in later stages in vivo (Fig. 7f and SI Appendix, Fig. S7a, b). Conclusively, the results demonstrated that PERK activation promoted TFEB nuclear translocation, thereby facilitating the two-phase regulation through TFEB’s transcriptional control.

### Modulating mitophagy influenced osteogenesis during retention and relapsing

**Fig. 8:**
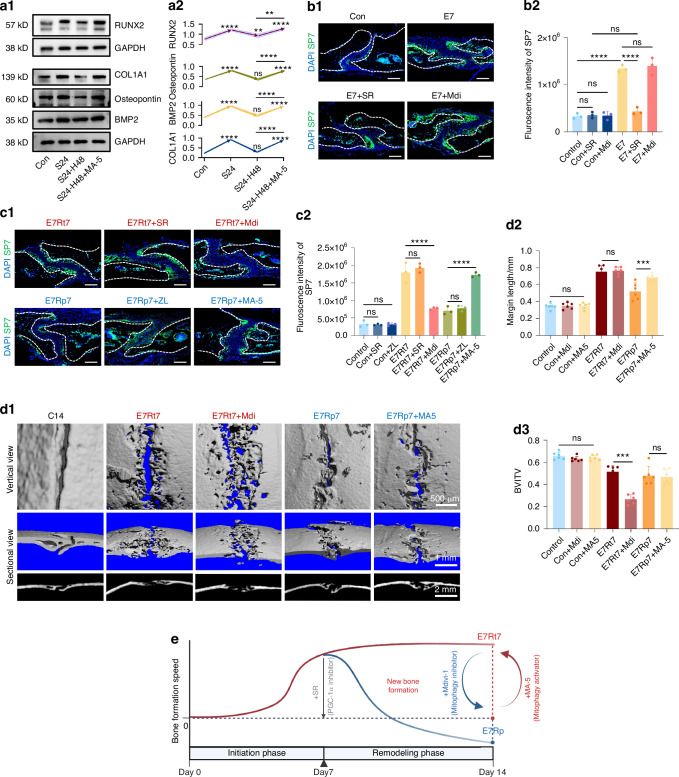
Regulation of osteogenesis and relapse progression via mitochondria manipulation. **a** Western-bolt analysis of RUNX2, COL1A1, Osteopontin and BMP2 expression in control (Con), 24-h stretch (S24), 24-h stretch followed by a 48-h halt (S24-H48), and H48 groups treated with the mitophagy activator MA-5 immediately after the cessation of stretch (S24-H48 + MA-5). *n* = 4/group. **b**, **c** Immunofluorescence staining of SP7 in suture areas. Scale bar: 100 µm. *n* = 4–6/group. **d** Morphological assessment of bone changes: **d1** Representative micro-CT images illustrating bone length and quality in the expansion-activated frontal region; **d2** Comparison of marginal bone length; **d3** Evaluation of bone volume fraction (BV/TV) ratios. *n* = 4–6/group. **e** Illustration depicting new bone formation along cranial suture expansion and relapse progression. Throughout the expansion phase, osteogenic activity steadily increased, peaking around the 7-day mark and maintaining during retention. However, upon relapse, osteogenesis shifted towards osteoclast activity, leading to subsequent bone loss. Treatment with SR-18292 (SR) during the initial 7-day period inhibited early bone formation, while Midvi-1 (Mdi) administration during retention impacted remodeling-associated bone formation. Conversely, the use of MA-5 partially mitigated bone loss resulting from relapse. Data are presented as mean ± standard deviation (SD). ns > 0.05, *P < 0.05, **P < 0.01, ***P < 0.001, ****P < 0.000 1

We then explored the potential of modulating osteogenesis and relapse progression by manipulating mitochondrial dynamics under mechanical force. Initially, introducing the mitophagy activator MA-5 into the culture medium after stretching cessation significantly sustained osteogenic potential, contrasting the expected decline after a 48-h break (Fig. 8a). To validate these findings in vivo, Gel-MA loaded with the PGC-1α activator ZL and inhibitor SR, as well as mitophagy inhibitor Mdivi-1 and activator MA-5, were employed to assess the impact of mitochondrial dynamics on osteogenesis, respectively. As anticipated, SR administration in the initial stage, rather than Mdivi-1, affected osteogenesis, confirming that osteogenic ability in this phase was governed by mitochondrial biogenesis and PGC-1α activation (Fig. 8b). Conversely, in the later stage, Mdivi-1 inhibited the expected osteogenic enhancement from retention, while MA-5 counteracted the diminished osteogenic capacity induced by relapse forces (Fig. 8c and SI Appendix, Fig. S8a, b). Morphologically, bone edge length was significantly reduced due to relapse compared to the retention group (Fig. 8d). Although Mdivi-1 did not significantly affect bone length in the retention group, it compromised new bone quality, as indicated by BV/TV ratios. Conversely, MA-5 preserved new bone formation, evidenced by the extended bone length compared to the relapse group. However, straightforward administration of MA-5 only partially mitigated the impact of relapse-induced forces and still resulted in impaired bone formation compared to the retention group (Fig. 8d). Conversely, the application of SR or ZL had no effect on either retention or relapse group (SI Appendix, Fig. S8c). Collectively, coordinated mitochondrial dynamics played a critical role in the growth and maintenance of osteogenic potential induced by mechanical stimuli. Specifically, mitochondrial biogenesis supported the initial phase, while timely clearance was essential in the later stage. During relapse, impaired mitophagy disrupted this balance, leading to the loss of osteogenesis and suboptimal bone formation (Fig. 8e).

## Discussion

**Fig. 9:**
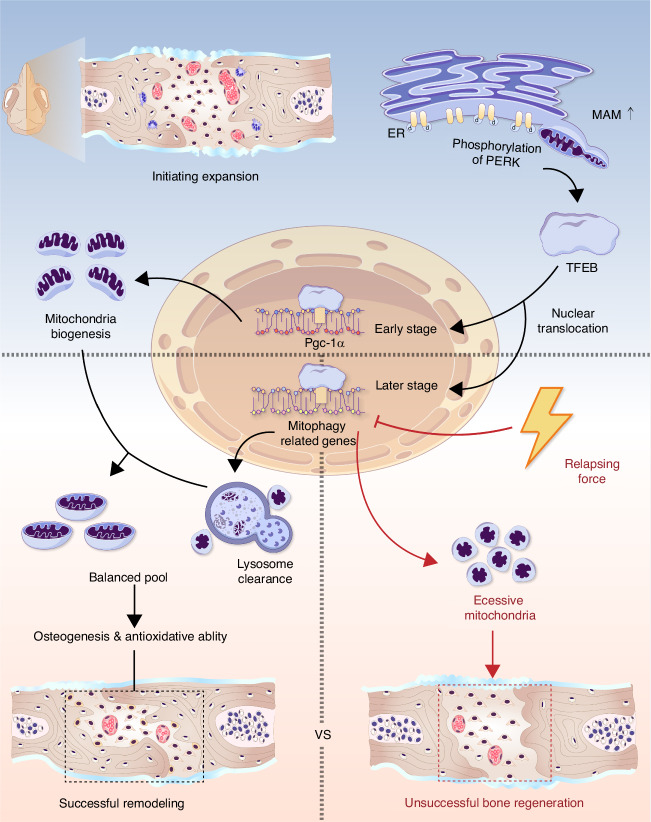
Schematic depiction summarizing the key findings of this study. Cranial suture expansion force induces endoplasmic reticulum (ER) stress and enhances ER-mitochondria contact. Under ER stress, phosphorylation of PERK—localized on mitochondria-associated ER membranes (MAM)—triggers the nuclear translocation of the transcription factor TFEB. TFEB activation proceeds in two stages: initially promoting the transcription of PGC-1α to enhance mitochondrial biogenesis, followed by upregulation of mitophagy-related genes to facilitate the clearance of excessive mitochondria. This coordinated, sequential regulation maintains mitochondrial homeostasis, supporting osteogenesis and antioxidative capacity during expansion and ultimately leading to successful bone remodeling. During relapse (illustrated by the red line), TFEB-mediated mitophagy is suppressed, resulting in delayed clearance of excessive mitochondria, accumulation of dysfunctional mitochondria, and impaired bone formation

This study investigated craniofacial suture expansion and relapse process, emphasizing the orderly biogenesis and subsequent clearance of mitochondria ensure the successful bone regeneration under mechanical stimuli, which is orchestrated sequentially by ER stress/p-PERK/TFEB signaling (Fig. 9). During successful remodeling, a significant increase in mitochondria count was observed after 7 days of expansion, followed by a gradual decline due to activated mitophagy during retention. Conversely, premature withdrawal of the expanding force led to insufficient mitophagy, resulting in mitochondria accumulation and impaired bone generation. This regulation of the crucial rise-and-fall cycle of mitochondria was initiated by ER stress activation due to mechanical strain, leading to PERK phosphorylation, which then promoted TFEB nuclear translocation for transcriptional activities. The two-stage effect was directly mediated by TFEB, which sequentially activated PGC-1α for biogenesis and mitophagy-related genes for clearance. Pharmaceutical activation of mitophagy partially rescued bone loss caused by relapse, while its inhibition hindered normal osteogenesis post-suture expansion. Overall, our findings elucidate the PERK-TFEB-mediated control of mitochondria biogenesis and clearance, ensuring effective oxidative ability and bone formation while reducing relapse during craniofacial suture expansion under mechanical strain.

The sequential regulation of mitochondria biogenesis and mitophagy activation mediated by TFEB is intriguing. TFEB, a member of the microphthalmia-associated transcriptional factor family, acts by directly binding to promoters of the coordinated lysosomal expression and regulation (CLEAR) element,^49^ serving as a master regulator of the autolysosome pathway. Its transcriptional activity is contingent upon nuclear localization.^50^ The mechanisms governing TFEB’s subcellular localization in response to various signals remain diverse and not fully elucidated. Previous studies have demonstrated that under ER stress conditions, the UPR triggers PERK/eIF2S1 phosphorylation, thereby controlling TFEB’s subcellular localization and activity, which motivated our investigation.^44^ The regulatory relationship between TFEB and PGC-1α merits attention. PGC-1α, a major nuclear-encoded transcriptional coactivator of mitochondrial biogenesis, can be directly bound by TFEB at its promoter.^48^ Conversely, PGC-1α is also capable of regulating TFEB.^51^ The mechanisms underlying this bidirectional regulation remain intriguing, but likely connect to the overall energy and metabolic state of the cell. In the craniofacial suture expansion model used in our study, the initial expansion phase emerges as a stress state, prompting rapid proliferation of MSCs.^8^ This energy-dependent cellular process may necessitate mitochondrial biogenesis to augment mitochondrial numbers to meet the energy demand.^10^ However, as the remodeling process progresses to the later stage, cellular energy demands shift, and the presence of excessive mitochondria could prove detrimental to cells, underscoring the necessity for timely activation of mitophagy. The binding transition of TFEB to diverse sequences, as shown in our study, may relied on the overall energy state, which warrant future study.

A noteworthy discovery in our research centered on the relationship between the ER and mitochondria and its advantageous role in mechanical osteogenesis. Previous studies have recognized the activation of ER stress in response to mechanical stimuli, focusing primarily on inflammatory contexts or supra-physiological forces,^22,25,26^ often concluding that elevated ER stress may induce cell apoptosis. However, adequate elevation of ER stress could contribute positively to cellular homeostasis,^23,44^ echoing by our study, whereas excessive or uncontrolled levels may indeed be detrimental. Similarly, the role of mitochondria under mechanical stimuli remains a topic of debate. While physiological forces are generally thought to enhance mitochondrial activity and support cellular homeostasis, supra-physiological or pathological forces may impair mitochondrial function, disrupting cellular balance.^27–36,52^ Notably, specific studies investigating the impact of mechanical forces on mitochondrial function in osteogenesis are still lacking. Our work identified the sequential changes of mitochondria pools was pivotal for MSCs to acquire antioxidative and osteogenic ability confronting force, rather than NRF2, the previously reported key regulator for oxidation resistance.^37^ Intriguingly, the bone resorption activities seemed to be impacted as well by modulating mitophagy. During expansion process as in both E7 and E7t7 groups, the resorption mainly happened around the marginal bone, which indicating the remodeling of new bone. Once impeding the mitophagy in the E7t7 group, the TRAP activities slightly increased in the suture area. Conversely, in the relapsing group, the suture area showed intense TRAP activities, which could be blocked by the mitophagy activation (SI Appendix, Fig. S9). However, the exact mechanism concerning osteoclastic process merits more exploration. Regarding the connections between these two organelles,^18–20,31^ prior evidence has not indicated a relationship between these organelles under mechanical forces in MSCs that participated in new bone formation yet. In our research utilizing mechanical stretching, we have identified that ER stress served as the pivotal upstream factor for activating mitophagy in MSCs. This precise coordination of the ER-mitochondria connection significantly contributed to the process of force-induced osteogenesis.

Clinically, midface and palatal bone hypodevelopment is often addressed through suture expansion. A crucial step in planning treatment is assessing the patient’s susceptibility to relapse, which depends on factors such as age, growth phase, and the degree of suture closure.^53^ For patients with low susceptibility, prolonged expansion may suffice to prevent relapse by promoting adequate new bone growth around the ridge. Conversely, for those with high susceptibility or those seeking shorter treatment durations, incorporating mitochondrial-regulating medications into the therapy may offer potential benefits. However, further clinical evidence is required to validate the efficacy and safety of such pharmaceutical interventions. Beyond pharmaceutical approaches, our study highlights that sublethal mitochondrial stressors, such as moderate mechanical stress on bone, can facilitate adaptation to excessive stress—a phenomenon known as mitohormesis.^54^ Consequently, regular and moderate exercise may benefit bone health, as mitohormesis represents a cytoprotective paradigm induced by mechanical stress. Nonetheless, the precise conditions under which mitohormesis applies and its extent require further investigation.

In conclusion, our findings demonstrate that PERK phosphorylation upon ER stress orchestrated mitochondrial dynamics via TFEB, facilitating both biogenesis and the timely removal of mitochondria through mitophagy. This coordination enhanced antioxidative capacity and osteogenic potential during mechanical stretching. Pharmacological modulation of mitophagy in vivo significantly impacts bone formation during both suture expansion and relapse processes, which provides delightful insight into clinical application.

## Materials and methods

A full description of the “Materials and methods” is provided in SI Appendix, including cell culture, in vivo and in vitro model construction, pharmaceutical treatment, flow cytometry, ROS measurements, electron microscopy, Western blot, ERTracker, MitoTracker and LysoTracker staining, CUT&RUN-qPCR, immunohistochemistry, immunofluorescence staining, confocal microscopy, calcium dying, microcomputed tomography (µCT) analysis and statistical analysis.

## Supplementary information

SI Appendix

## Data Availability

All data are available in the main text or the Supplementary Materials.
