# Supplementary material for: ER-induced PERK/TFEB cascade sequentially modulates mitochondrial dynamics during cranial suture expansion
Source: Bone Res. 2025 Jun 23;13:66. doi: 10.1038/s41413-025-00427-y (PMC12185732; doi:10.1038/s41413-025-00427-y)
Supplement: Supplementary file 1 — SI Appendix [file 41413_2025_427_MOESM1_ESM.docx]

**Supporting Information for**

**ER-induced PERK/TFEB cascade sequentially modulates mitochondrial dynamics during cranial suture expansion**

**Jingyi Cai,^1^ Ziyang Min,^1^ Chaoyuan Li,^3^ Zhihe Zhao,^1^* Jun Liu,^1^* Dian Jing^2^***

^1^ State Key Laboratory of Oral Diseases & National Clinical Research Center for Oral Diseases, West China Hospital of Stomatology, Sichuan University, Chengdu, 610041 China

^2^ Department of Orthodontics, Shanghai Ninth People’s Hospital, Shanghai Jiao Tong University School of Medicine; College of Stomatology, Shanghai Jiao Tong University; National Center for Stomatology; National Clinical Research Center for Oral Diseases; Shanghai Key Laboratory of Stomatology, Shanghai, 200011, China

^3^ Department of Implantology, School and Hospital of Stomatology, Shanghai Engineering Research Center of Tooth Restoration and Regeneration, Tongji University, Shanghai, China

***Correspondence:**

Jun Liu, Email: junliu@scu.edu.cn; Zhihe Zhao ([zhzhao@scu.edu.cn](mailto:zhzhao@scu.edu.cn)); Dian Jing, Email: [jingdian@shsmu.edu.cn](mailto:jingdian@shsmu.edu.cn);

**This PDF file includes:**

Supporting text

Figures S1 to S9

SI References

Supporting Information Text

**Supplementary Information for Method and Material**

**Cell Culture and Stretching Application**

C3H10T1/2 cells were procured from Procell Inc. and maintained in αMEM (Gibco) supplemented with 10% fetal bovine serum (Gibco) and 1% penicillin-streptomycin (Gibco) at 37°C in a humidified atmosphere containing 5% CO2. Mechanical cyclical stretch was applied to the cells using the Flexcell FX-5000™ Tension System (Flexcell International Corporation). Prior to stretching, cells were seeded onto Rat Tail Collagen Type I (Corning)-coated BioFlex silicon plates (Flexcell International Corporation). After a 12-hour adhesion period, the cells underwent cyclical mechanical stimulation in the Flexcell FX-5000™ Tension System at 10% elongation and a frequency of 0.5 Hz for durations of 1, 6, and 24 hours. Control cells were cultured under identical conditions but were not subjected to the stretch protocol.

**Pharmaceutical Intervention**

The powder was initially dissolved in DMSO to prepare a stocking solution, which was then stored at -80°C. The final concentration used *in vitro* and *in vivo* were listed in Table 1. Before use, the stocking solution was added to the medium, resulting in a final DMSO concentration of 0.04%. Different medication timings *in vitro* were selected for various purposes:

1. For continuous application, medication was added to the medium 2 hours before stretching.

2. For mid-application, cells were initially stretched for 6 hours, then quickly transferred to the operating bench, where medication was added to the medium. Subsequently, cells were resumed under stretch force.

3. For post-application, medication was added immediately after stretching, and the cells were returned to the incubator for 24 hours.

| Product Name | Catalog | Concentration *in vitro* | Concentration *in vivo* |
| --- | --- | --- | --- |
| 4-Phenylbutyric acid (4-PBA) | S3592, Selleck | 20mM |  |
| 2-Aminoethyl diphenylborinate | CSN23287, CSNpharm | 15mM |  |
| Mdivi-1 | CSN16979, CSNpharm | 5mM | 10mg/kg |
| Mitochonic acid 5 (MA-5) | CSN21177, CSNpharm | 10mM | 15mg/kg |
| GSK2606414 | CSN15902, CSNpharm | 10mM | 10mg/kg |
| Tfeb inhibitor | CSN21986, CSNpharm | 10mM | 10mg/kg |
| Tfeb activator 1 | CSN25353, CSNpharm | 10mM |  |
| ZLN005 | CSN15985, CSNpharm | 15mM | 20mg/kg |
| SR-18292 | CSN19255, CSNpharm | 10mM | 20mg/kg |

### Table S1. Concentration list of Pharmaceutical Intervention

**Animals and Treatments**

Four-week-old male C57 mice were obtained from Dashuo (China) and housed in cages with a 12-hour light/dark cycle. All animal procedures were conducted in compliance with the ethical standards of the institution and approved by the Institutional Animal Care and Use Committee of Sichuan University West China Hospital of Stomatology (WCHSIRB-D-2019-128). The cranial suture expansion model was established as previously described (1). Briefly, mice were anesthetized with a combination of ketamine (80 mg/kg) and xylazine (10 mg/kg), and the surface of the calvaria bone was exposed after removing fur. Holders were cemented 3 mm from the sagittal suture using a light-cured adhesive (Unitek, 3M). After allowing 2 days for skin recovery, a tensile force of 25 ± 5 g was applied using springs. Sham-operated control groups underwent the same procedure without force application. For in vivo intervention, Gel-MA (EFL-GM-30, EngineeringForLife) powder was first dissolved in pre-warmed PBS to achieve a final concentration of 7%. Before use, Gel-MA was heated in a warm water bath until it turned into a liquid form and mixed with the medication stock solution. The hydrophobic chemicals were initially mixed with 1% F-127 (EFL-F127DA-001, EngineeringForLife) to enhance dispersibility in Gel-MA. The final concentrations of medications are listed in Table 1. A total of 40 μL of the mixed solution was subcutaneously injected into the cranial suture region and solidified using curing light. Injections were performed every 7 days.

**Western Blot**

Cell samples were lysed using RIPA buffer (Beyotime) to obtain protein extracts, followed by boiling at 95°C for 10 min in 1× loading buffer (Beyotime), and subjected to SDS-PAGE (Biorad). The proteins were transferred onto PVDF membranes, blocked with 5% dry milk in TBST buffer, and probed with primary antibodies, including GAPDH (1:5000, ET1601-4, Huabio), LAMP1 (1:2000, ab208943, Abcam), DRP1 (1:1000, ab184247, Abcam), PGC-1α (1:1000, A12348, ABclonal Technology), Parkin (1:1000, ET1702-60, Huabio), PINK1 (1:1000, A7131, ABclonal Technology), SOD2 (1:2000, ET1701-54, Huabio), TOMM20 (1:2000, ET1609-25, Huabio), COX IV (1:1000, ET1701-63, Huabio), VDAC1 (1:1000, ET1601-20, Huabio), NRF2 (1:1000, 12721, Cell Signaling), p-NRF2 (1:1000, ET1608-28, Huabio), FIS1 (1:1000, A19666, ABclonal Technology), PERK (1:1000, ER64553, Huabio), p-PERK (1:1000, 3179, Cell Signaling), eIF (1:1000, 5324, Cell Signaling), p-eIF (1:1000, 3398, Cell Signaling), ATF4 (1:1000, 11815, Cell Signaling), XBP1s (1:1000, A22546, ABclonal Technology), LC3A/B (1:1000, 12741, Cell Signaling), RUNX2 (1:1000, ab192256, Abcam), Osteopontin (1:1000, ER1802-16, Huabio), COL1A1 (1:1000, HA722517, Huabio), BMP2 (1:1000, PA5-85956, Invitrogen) and TFEB (1:1000, A7311, ABclonal Technology). After washing three times in TBST buffer, the membranes were incubated with goat anti-rabbit IgG-HRP secondary antibodies (1:10000, HA1001, Huabio) for 1 h at room temperature, followed by three additional washes in TBST buffer. Chemiluminescent signals were detected using SuperKine™ Universal ECL Substrate (Abbkine, BMP3010).

**Cellular Immunofluorescent Analyses**

Following treatment, cells were fixed with 4% paraformaldehyde and permeabilized with 0.1% Triton X-100. Nonspecific binding was blocked with 5% normal goat serum in PBS. The bottom surface of BioFlex silicon plates (Flexcell International Corporation) was cut into small pieces and placed into confocal dishes for antibody incubation. Slides were then probed with primary antibodies in blocking buffer overnight at 4°C. The antibodies used included p-NRF2 (1:100, ET1608-28, Huabio), FIS1 (1:100, A19666, ABclonal Technology), ATF4 (1:100, 11815, Cell Signaling), XBP1s (1:50, A22546, ABclonal Technology), TFEB (1:100, A7311, ABclonal Technology), TOMM20 (1:400, ET1609-25, Huabio), and Alexa Fluor® 647 Rabbit monoclonal to LAMP1 (1:500, EPR21026, Abcam). Subsequently, slides were treated with Goat anti-Rabbit IgG (H+L) Cross-Adsorbed Secondary Antibody, Alexa Fluor™ 488 (1:2000, A-11008, Invitrogen) for 1 h at room temperature. Finally, slides were washed and stained with DAPI for 10 min. Mounted slides were visualized using a laser scanning confocal microscope (FV3000, Olympus). All representative images were taken under the same exposure conditions for each condition.

***In vivo*** **Immunofluorescent Analyses**

Cranial bones were dissected and fixed in 4% paraformaldehyde overnight at 4°C, followed by decalcification in 10% EDTA (pH 7.4) for 3-5 days at 4°C. For immunofluorescence section, after dehydration in 15% and 30% sucrose solutions for 1 day each at 4°C, samples were embedded in OCT (Sakura Tissue-Tek) and sectioned at 10 μm using a cryosectioning machine (CM1950, Leica). Sections were washed with 0.5% Triton X-100 in PBS for 10 minutes, followed by three PBS washes. Subsequently, sections were digested with hyaluronidase from bovine testes (H4272, Sigma-Aldrich) in CAPSO buffer (C2278, Sigma-Aldrich) (10mM). After three washes, sections were incubated in blocking buffer (5% BSA and 10% goat serum in PBS) for 1 h at 37°C and then stained with primary antibodies in blocking buffer overnight at 4°C. The primary antibodies used were SP7 (1:400, ab209484, Abcam), Parkin (1:50, ET1702-60, Huabio), TOMM20 (1:400, ET1609-25, Huabio), FIS1 (1:200, A19666, ABclonal Technology), BMP2 (1:200, PA5-85956, Invitrogen) and RUNX2 (1:200, ab192256, Abcam). Slides were washed three times with PBS and then stained with Goat anti-Rabbit IgG (H+L) Cross-Adsorbed Secondary Antibody, Alexa Fluor™ 488 (1:2000, A-11008, Invitrogen) in blocking buffer for 1 h at room temperature. Slides were then washed three times with PBS and mounted with enhanced antifade mounting medium with DAPI (SuperKine). Images were acquired using a laser scanning confocal microscope (FV3000, Olympus). All representative images were taken under the same exposure conditions for each condition.

**Organelle Labeling in Live Cells**

After the stretching procedure, cells were treated with media containing organelle-selective dyes. Specifically, the cells were exposed to endoplasmic reticulum-selective ERTracker™ Green dye (E34251, Invitrogen), mitochondrion-selective MitoTracker™ Dyes for Mitochondria Labeling (M22426, Invitrogen), and lysosome-selective LysoTracker Green (L7526, Invitrogen) for 20 minutes in the cell incubator. Subsequently, fluorescence signals were visualized using a confocal microscope (FV3000, Olympus).

**ROS Measurements and Flow cytometry**

Intracellular ROS levels were evaluated using DCFH-DA (S0033S, Beyotime) by treating cells with 10 mM DCFH-DA at 37°C for 20 minutes, followed by two washes with PBS. Analysis was performed using flow cytometry (BD Biosciences).

**Transmission Electron Microscope**

Cells were digested and centrifuged after stretching. The cell precipitate was fixed in 2.5% glutaraldehyde for two hours at 4°C, post-fixed with osmic acid, dehydrated, embedded, and sectioned. After double staining with lead citrate and uranyl acetate, images were acquired using a TEM system (HT7800, Hitachi, Japan).

**Cellular Calcium Dying**

Mag-Fluo-4 AM (20401, AAT Bioquest) and Rhod-2, AM (40776ES50, Yeasen) were utilized for ER and mitochondria calcium staining, respectively. Working solutions of 5 µM were prepared by diluting 5 mM stock solutions in DMSO with Hanks and Hepes buffer containing 0.04% Pluronic F-127 (P6790, Solarbio). After stretching, the medium was replaced with fresh HHBS buffer, and cells were incubated with Mag-Fluo-4 AM working solution at 37°C for 30 minutes. Excess probes were subsequently removed by incubating cells in HHBS at 37°C for 20 minutes. [Ca2^+^] mito and [Ca2^+^]ER were simultaneously measured using 490 nm and 550 nm excitation wavelengths.

**CUT&RUN qPCR**

The CUT&RUN was carried out according to the protocol of the Hyperactive pG-MNase CUT&RUN Assay Kit for PCR/qPCR (HD101-01, Vazyme). Specifically, immediately after stretch, cells were first digested with trypsin (Gibco) and counted. 4X10^5^ cells were collected for each sample for following experiment. The TFEB antibody (1:50, 83010, Cell Signaling) was pre-incubated with ConA Beads Pro and then incubated with cells in 4°C overnight. After washes, beads were incubated with pG-MNase enzyme with rotation at 4°C for 1 hour. Then CaCl_2_ was added for fragmentation on ice for 90 minutes. Then the stop buffer was added with Spike in DNA at 37°C for 30 minutes. DNA was then extracted with column and qPCR was applied with primers below.

Spike in DNA sequence:

ATAACTCAATGTTGGCCTGTATAGCTTCAGTGATTGCGATTCGCCTGTCTCTGCCTAATCCAAACTCTTTACCCGTCCTTGGGTCCCTGTAGCAGTAATATCCATTGTTTCTTATATAAAGGTTAGGGGGTAAATCCCGGCGCTCATGACTTCGCCTTCTTCCCATTTCTGATCCTCTTCAAAAGGCCACCTGTTACTGGTCGATTTAAGTCAACCTTTACCGCTGATTCGTGGAACAGATACTCTCTTCCATCCTTAACCGGAGGTGGGAATATCCTGCATTCCCGAACCCATCGACGA

| Gene Name | Forward Primer | Reverse Primer |
| --- | --- | --- |
| *Lamp1* | CAGCACTCTTTGAGGTGAAAAAC | ACGATCTGAGAACCATTCGCA |
| *Prkn* | TCTTCCAGTGTAACCACCGTC | GGCAGGGAGTAGCCAAGTT |
| *Ppargc1a* | TATGGAGTGACATAGAGTGTGCT | CCACTTCAATCCACCCAGAAAG |

**Table S2. qPCR primer sequence**

**Microcomputed Tomography (µCT) Analysis**

Calvarial bones were fixed in 4% PFA at 4°C overnight and then stored in 0.5% PFA before scanning. Samples were scanned using a micro–computed tomography (μCT) 35 imaging system (Scanco Medical, Bruttisellen, Switzerland) with a voltage of 70 kV and a voxel size of 6 μm. Analysis of bone structural parameters was performed using Scanco Medical software, with a threshold of 220–1000 utilized to segment mineralized bone from air and soft tissues. The length of marginal bones was measured between three points located on the top, middle, and base areas of each sample.

**Statistical Analysis**

All experiments were performed independently at least three times, and the results are expressed as mean ± standard deviation. Statistical comparisons between two groups were conducted using unpaired two-tailed Student's t-tests with GraphPad Prism 9 (USA). For comparisons involving more than two groups, one-way ANOVA with Tukey's test was utilized. P values less than 0.05 were considered statistically significant (*P < 0.05, **P < 0.01, ***P < 0.001).

**References**

1. D. Jing, *et al.*, Response of Gli1(+) Suture Stem Cells to Mechanical Force Upon Suture Expansion. *Journal of bone and mineral research : the official journal of the American Society for Bone and Mineral Research* **37**, 1307–1320 (2022).

Supplementary Figures

**Fig. S1. (a)** Flow cytometry analysis of cellular ROS levels via DCFH-DA detection in control (Con) and 1, 6, 24-hour stretch group. **(b)** Ratio of phosphorylated NRF2 (p-NRF2) to NRF2 confronting stretch. N=3/group. **(c)** NRF2 nuclear localization after 24-hour stretch. N=6/group. Scale bar: 10 µm. Data are presented as mean ± standard deviation (SD). ns > 0.05, *p < 0.05, **p < 0.01, ***p < 0.001, ***p < 0.001.

**Fig. S2. (a)** Western blot results of the ratio of p-PERK/PERK, p-eIF2/eIF2α, and expression level of XBP1s in response to mechanical stretch. N=3/group. **(b-c)** Immunofluorescence analysis of activating transcription factor 4 (ATF4) and X-box-binding protein 1 (XBP1s) after 6- and 24-hour stretch. Scale bar: 10 µm**.** N=9/group. **(d)** Flow cytometry results of cellular ROS levels via DCFH-DA detection in control (Con), control added with 4-phenylbutyric acid (Con+4-PBA), 24 hour-stretch (Stre), stretch added with 4-PBA (Stre+4-PBA) groups. N=3/group. Data are presented as mean ± standard deviation (SD). ns > 0.05, *p < 0.05, **p < 0.01, ***p < 0.001, ***p < 0.001.

**Fig. S3.** **(a)** Co-staining of the ER Ca2+ dye (green) with the ER-tracker (red). **(b)** Co-staining of the mitochondria Ca2+ dye (red) with the Mito-tracker (green). Scale bar: 10 µm

**Fig. S4.** **(a)** Immunofluorescence staining of Fis1 in suture areas during expansion-relapse. Insets indicated by yellow squares. Red dotted line in a2 show comparison between E7d and E7t7. Scale bar: 100 µm (low), 25 µm (high). N=4-6/group. Data presented as mean ± SD. ns > 0.05, *p < 0.05, **p < 0.01, ***p < 0.001.

**Fig. S5.** **(a)** Immunofluorescence staining and statistical analysis of Parkin in the suture area post-intervention. Scale bar: 100 µm (low), 25 µm (high). N=3/group. Data presented as mean ± SD. ns > 0.05, *p < 0.05, **p < 0.01, ***p < 0.001.

**Fig. S6.** Immunofluorescence staining and statistical analysis of FIS1 in the suture area following GSK2606414 (GSK) intervention in 7-day expansion (E7) groups, as well as in 7-day expansion and another 7-day retention (E7Rt7) groups. Scale bar: 100 µm for low magnification and 25 µm for high magnification. N=3/group. Data are presented as mean ± standard deviation (SD). ns > 0.05, *p < 0.05, **p < 0.01, ***p < 0.001, ***p < 0.001.

**Fig. S7. (a-b)** Immunofluorescence staining and statistical analysis of FIS1 (a), and Parkin (b) in the suture area following TFEB inhibitor (Ti) intervention in 7-day expansion (E7) groups, as well as in 7-day expansion and another 7-day retention (E7Rt7) groups. Scale bar: 100 µm for low magnification and 25 µm for high magnification. N= 3/group. Data are presented as mean ± standard deviation (SD). ns > 0.05, *p < 0.05, **p < 0.01, ***p < 0.001, ***p < 0.001.

**Fig. S8.(a-b)** Immunofluorescence staining and statistical analysis of BMP2 (a), and RUNX2 (b) in the suture area following chemical intervention modulating mitophagy in retention (E7Rt7) and relapsing (E7Rp7) groups. Scale bar: 100 µm. N= 3/group. Data are presented as mean ± standard deviation (SD). ns > 0.05, *p < 0.05, **p < 0.01, ***p < 0.001, ***p < 0.001. **(c)** Representative micro-CT images (c1) and H&E staining images (c2) illustrating bone changes in the expansion-activated frontal region after SR and ZL application in retention group and relapsing group, respectively. Scale bars are labeled on the images.

**Fig. S9.** TRAP staining in the suture area following chemical intervention modulating mitophagy in retention (E7Rt7) and relapsing (E7Rp7) groups. Scale bar: 100 µm.
